# 

**Published:** 1895-09

**Authors:** 


### CONTENTS.

ORIGINAL ARTICLES:

Experiments with Tuberculin on Non-tuberculous Cows. By JAMES LAW, F.R.C.V.S. . . . . 537

Report on Vital Statistics, Virginia State V. M. A. By E. P. NILES, D.V.M. . 553

Address. By Dr. W. H. HARBAUGH, President Virginia State V. M. A. . . 561

A Suggestion or Aid to the Eradication of Tuberculosis by a Practical and Profitable Method. By A. S. HEATH, M.D., V.S. . . . . 573

Necrology . . . . . 576

REPORTS OF CASES:

I. Hæmatosalpinx and Hæmatometra in a St. Bernard Dog. II. Pyosalpinx in a Dog; Autopsies. By Dr. SPENCER . . . . . 577

Unusual Complication Attending Septicæmia Following Parturition; Autopsy. By M. O'M. KNOTT, V.S. . . . . 578

Imperforate Anus with False Opening: Anus Vulvalis. By J. T. DUNCAN, M.D., V.S. 580

I. Fracture of the Trapezium, or Rupture of One of the Tendons Attached to that Bone. II. Peritonitis. By Drs. MARSHALL and DIXON . . . . 582

The Use of Pyoctanin in Treatment of Quittors. By Dr. WILFRIED LELLMANN 583

CORRESPONDENCE . . . . . 586

LEGISLATION—Massachusetts—New York State Board of Medical Examiners . 593–597

EDITORIALS . . . . . 602

PERSONALS . . . . . 603

SOCIETY MEETINGS AND PROCEEDINGS:

U. S. V. M. A.—Keystone Veterinary Medical Association—Pennsylvania Alumni Association of the American Veterinary College—Wyoming Valley Veterinary Medical Association—Missouri State Veterinary Medical Association—Pennsylvania State Veterinary Medical Association—New York State Veterinary Medical Association—Iowa State Veterinary Meeting—Wisconsin Society of Veterinary Graduates—California State Veterinary Medical Association . 604–609

AMONG THE COLLEGES:

Ohio Veterinary College—Ontario Veterinary College—Veterinary Department, Detroit College of Medicine—National Veterinary College—Chicago Veterinary College—American Veterinary College . . . . . 609–611

